# Supplementary figures and images for: White matter microstructure in habit and reward circuits in anorexia nervosa: Insights from a neurite orientation dispersion and density imaging study
Source: Acta Psychiatr Scand. 2022 Nov 24;147(2):134–44. doi: 10.1111/acps.13521 (PMC9852024; doi:10.1111/acps.13521)

**Supplementary Table 1**: ANCOVA model


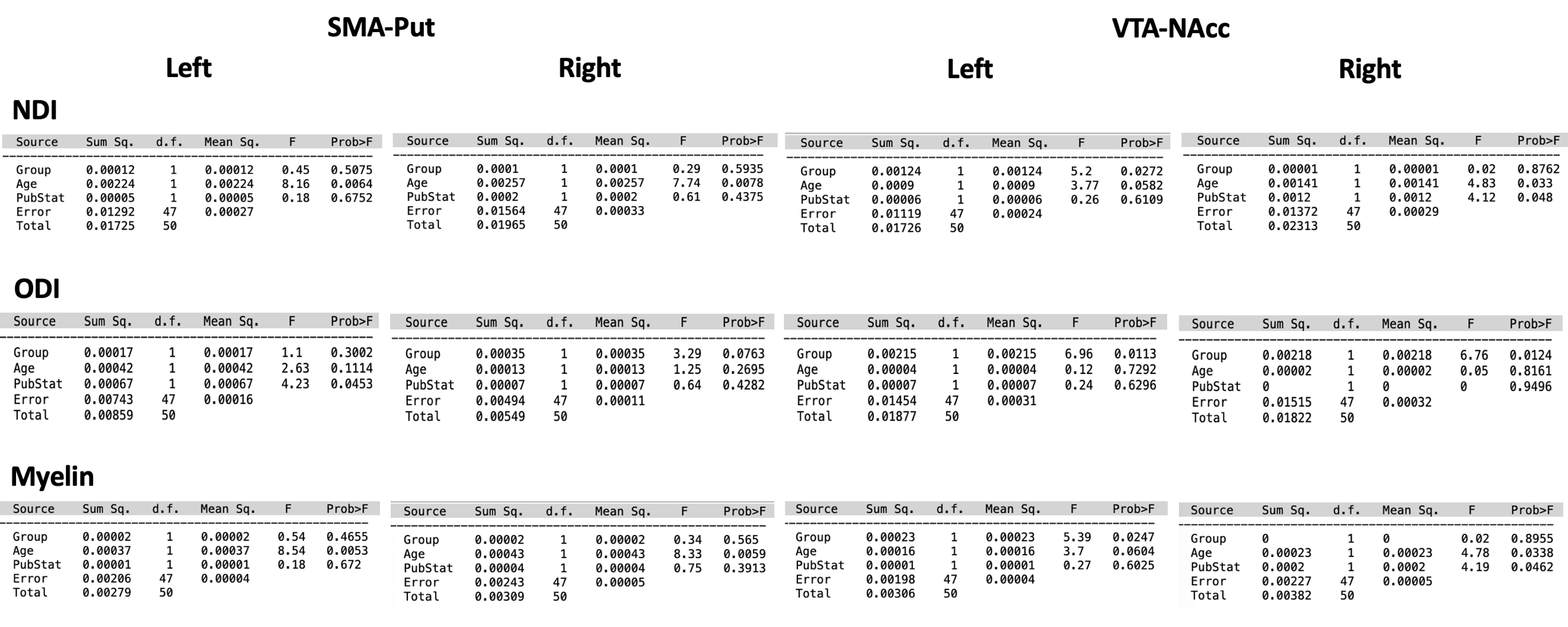

Supplement: Supplementary file 1 — Supplementary Table 1: ANCOVA model [file ACPS-147-134-s001.docx]
